# Supplementary material for: A programmable seekRNA guides target selection by IS1111 and IS110 type insertion sequences
Source: Nat Commun. 2024 Jun 19;15:5235. doi: 10.1038/s41467-024-49474-9 (PMC11187229; doi:10.1038/s41467-024-49474-9)
Supplement: Supplementary file 1 — Supplementary Information [file 41467_2024_49474_MOESM1_ESM.pdf]

Supplementary Information

A programmable seekRNA guides target selection by IS*1111* and IS*110* type insertion sequences

Rezwan Siddiquee[1], Carol H. Pong[1], Ruth M. Hall[1*], Sandro F. Ataide[1*]

[1]School of Life and Environmental Sciences

*Corresponding authors: Sandro F. Ataide sandro.ataide@sydney.edu.au and Ruth M. Hall ruth.hall@sydney.edu.au

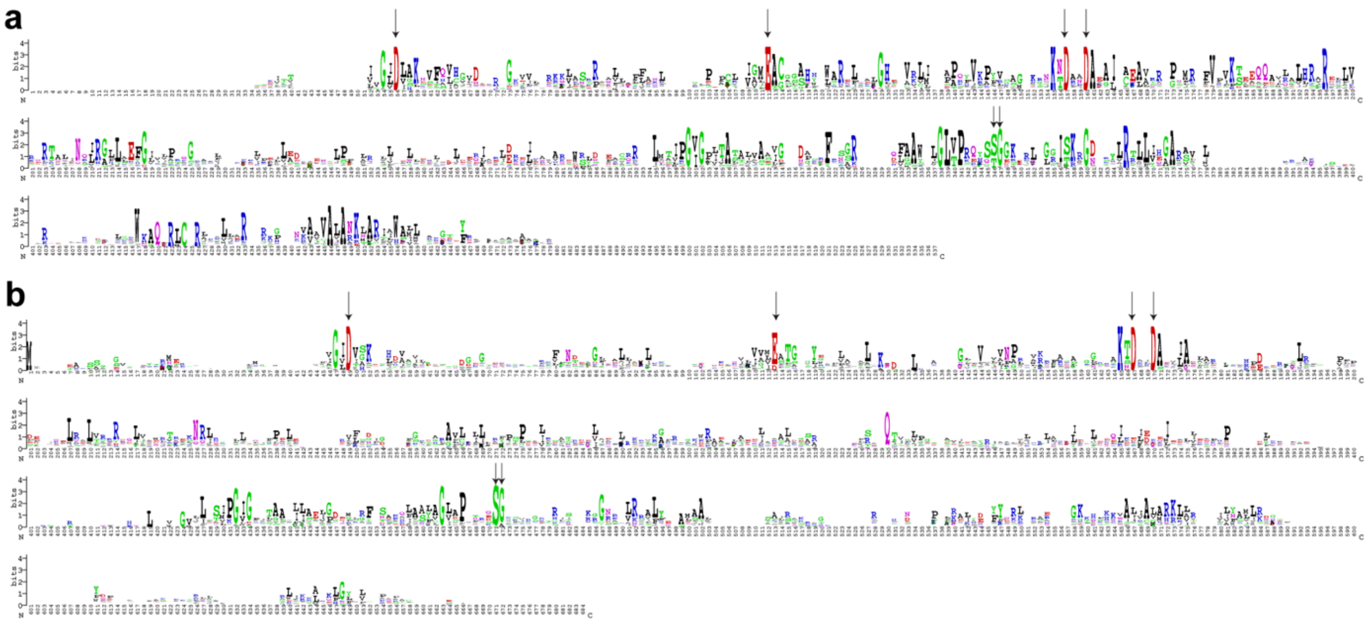

Supplementary Fig. 1: Conserved residues of the transposases from IS*110* and IS*1111* ISs aligned separately.

Transposase sequences from **a** IS*1111* family ISs and **b** IS*110* family ISs curated from ISfinder were aligned with Clustal Omega tool in MEGA and used to generate a Weblogo of sequence conservation. The arrows indicate the DEDD catalytic residues of the RuvC domains and SG motif in the C-terminal domains. Residues are coloured according to their chemical properties: polar (green), basic (blue), acidic (red), hydrophobic (black). Source data are provided as a Source Data file.

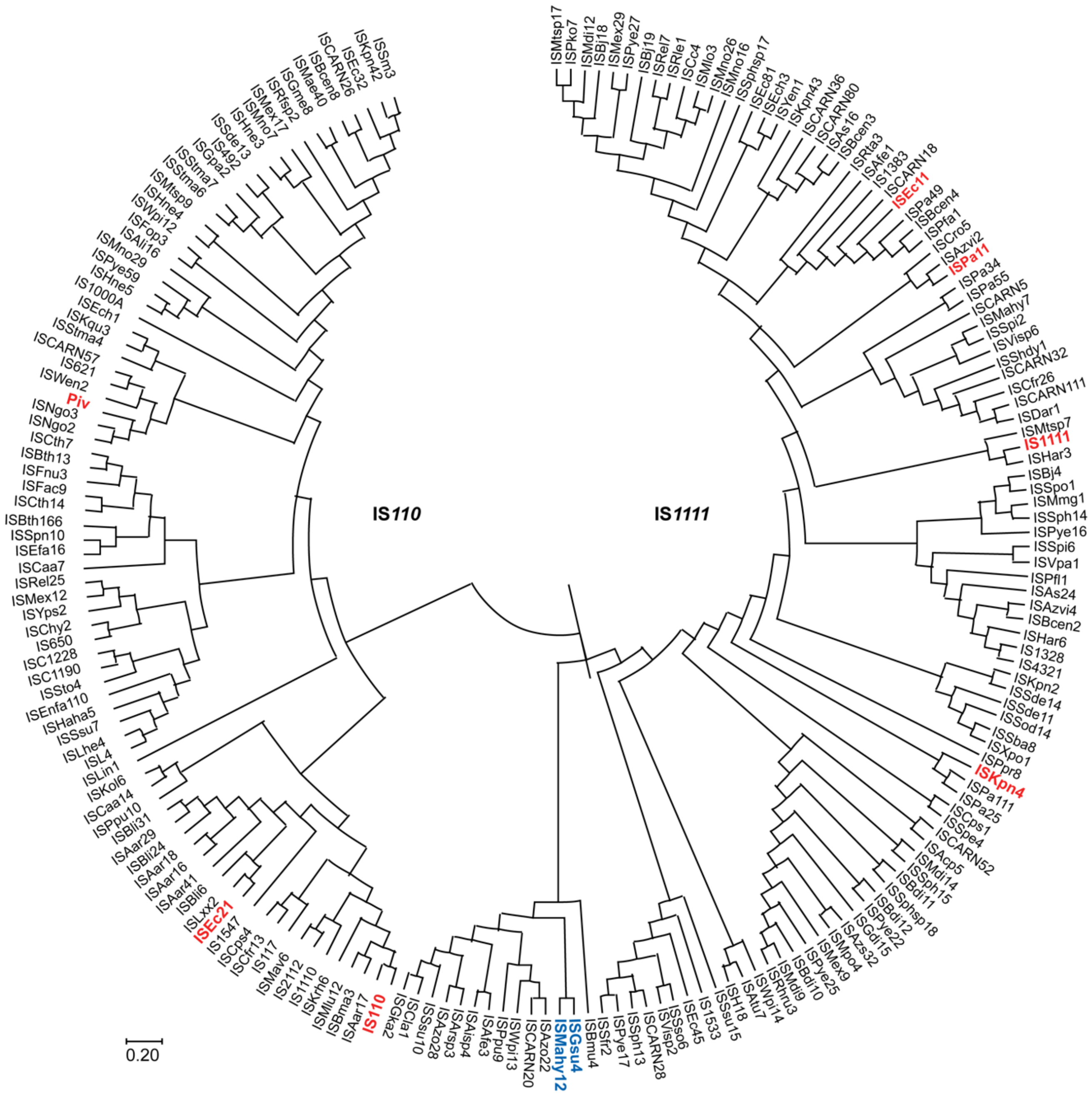

Supplementary Fig. 2: Phylogenetic tree of transposase sequences from members of IS*110* and IS*1111* families

Phylogenetic tree shown as a circle was generated on MEGA using a maximum likelihood neighbour-joining tree default settings and rooted using the midpoint. The MSA used to generate the tree is comprised of 196 sequences curated from the ISFinder to include a single representative of each cluster with >70% sequence identity. The sequence of the Piv inversion protein of *Moraxella lacunata* was included. First family members (IS*110* and IS*1111*), Piv and the ISs used in this study are shown in red. The ISs from the IS*1111* side shown in blue are exceptions that do not include IS*1111* family features. Source data are provided as a Source Data file.

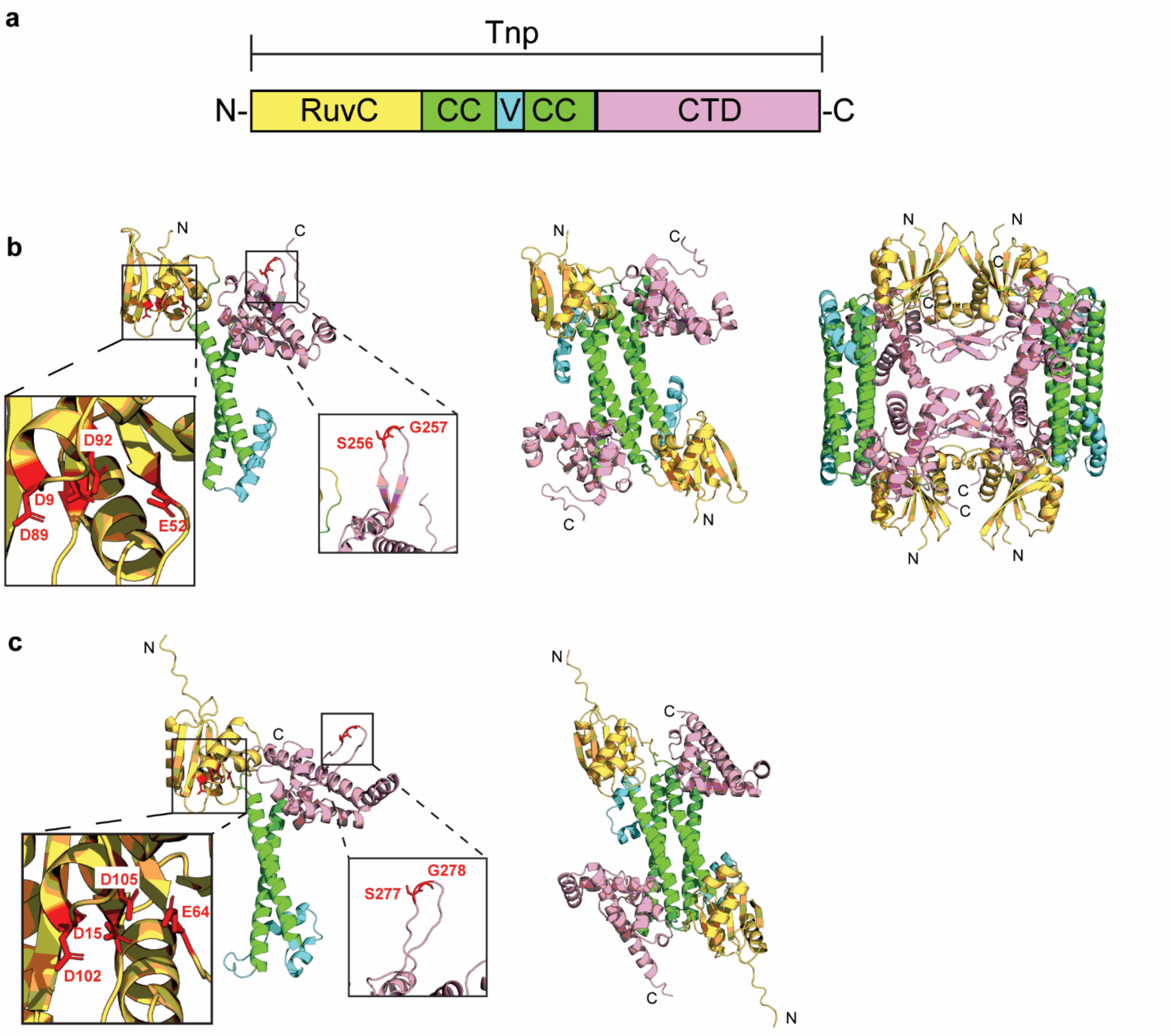

Supplementary Fig. 3: Alphafold2 structure prediction of transposases of IS*1111* and IS*110* IS.

a IS domain arrangement of IS*110* and IS*1111* family members. **b** Alphafold2 models of TnpEc11. Left panel – the monomer with the conserved catalytic amino acids DEDD from the N-terminal domain and SG from the C-terminal domain shown in red sticks. Middle panel - Alphafold2 dimer prediction driven by the coiled-coil interactions, placing the variable domain from one monomer next to the RuvC domain of the second monomer. Right panel – Alphafold2 tetramer prediction. **c** Alphafold2 models of TnpEc21. Left panel - monomer with the conserved catalytic amino acids DEDD from the N-terminal domain and SG from the C-terminal domain shown in red sticks. Middle panel - Alphafold2 dimer prediction driven by the coiled-coil interactions, placing the variable domain of a monomer next to the RuvC domain of the second monomer. Domain s in the structures are colour-coded: RuvC in yellow, coiled-coil (CC) in green, variable region (V) in cyan and C-term in pink.

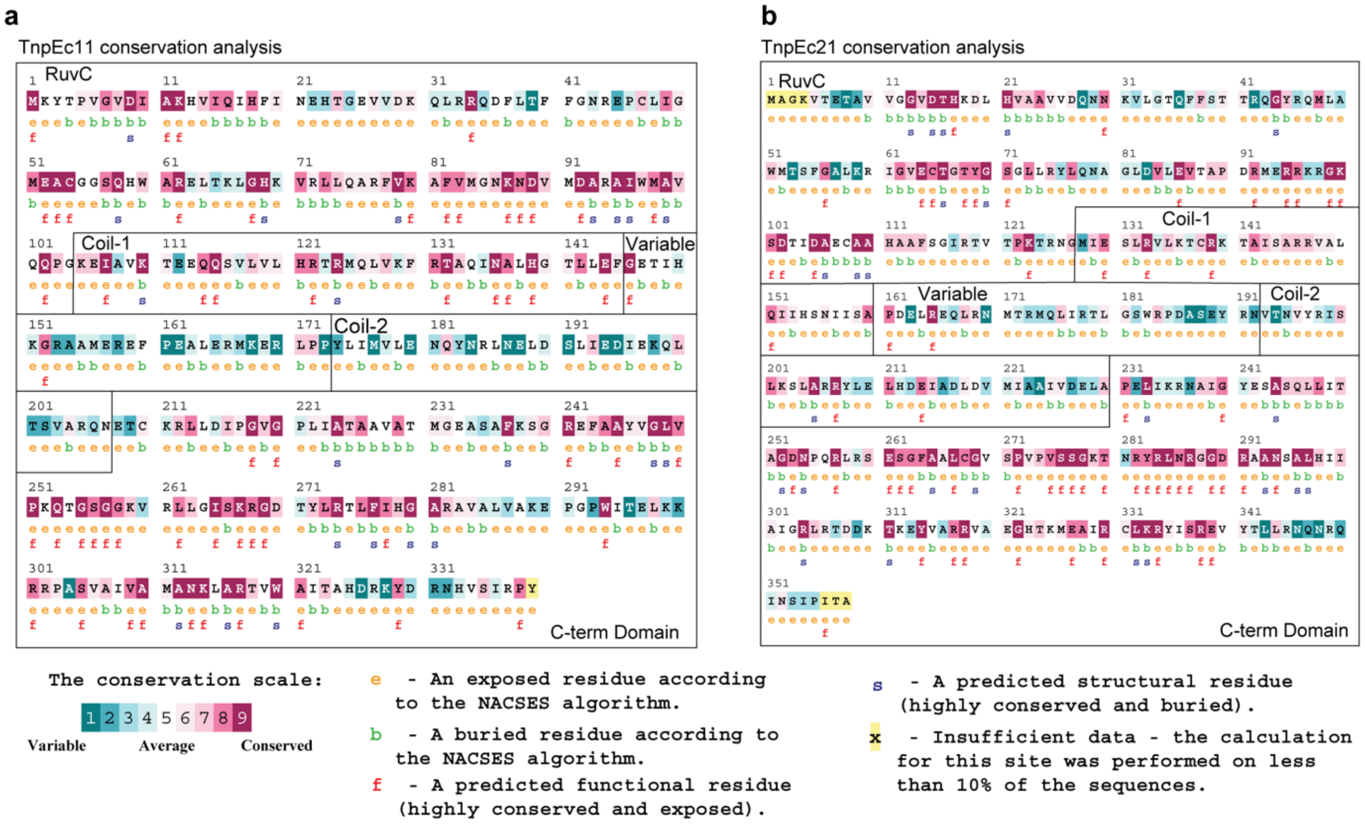

Supplementary Fig. 4: Transposase sequence conservation analysis of ISEc11 and ISEc21

a, ConSurf analysis of TnpEc11 as an example of IS*1111* IS with 150 sequences aligned. The transposase domains are separated with boxes. Conservation range is indicated on the right. Highly conserved residues are located on the RuvC and C-terminal domains. **b**, ConSurf analysis of TnpEc21 as an example of IS*110* IS with 120 sequences aligned. The transposase domains are separated with boxes. Conservation range is indicated on the right. Highly conserved residues are located on the RuvC and C-terminal domains.

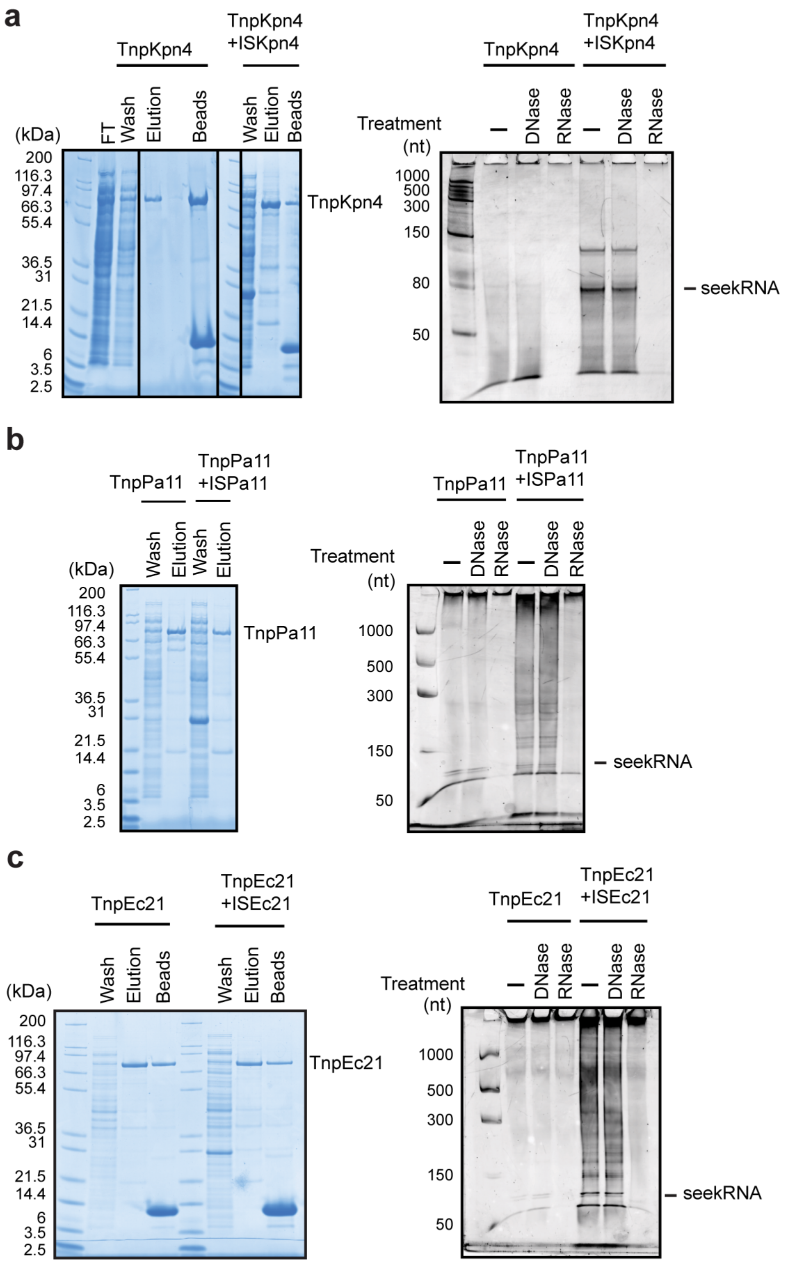

Supplementary Fig. 5: SDS-PAGE and nuclease digest gel analysis

a TnpKpn4 (85.7 kDa); **b** TnpPa11 (83.9 kDa); **c** TnpEc21 (86 kDa) purified alone or in the presence of their corresponding ISs (see Methods and Fig. 2). Left panels - Coomassie stained SDS-PAGE gels showing size and purity of fusion transposases containing an N-terminal 6X-His-MBP tag, and C-terminal StrepTag(II). Right panels - Post-purification, protein samples were digested with DNase or RNase and resolved in a 7 M Urea polyacrylamide denaturing gel and stained with SYBR GOLD. Source data are provided as a Source Data file.

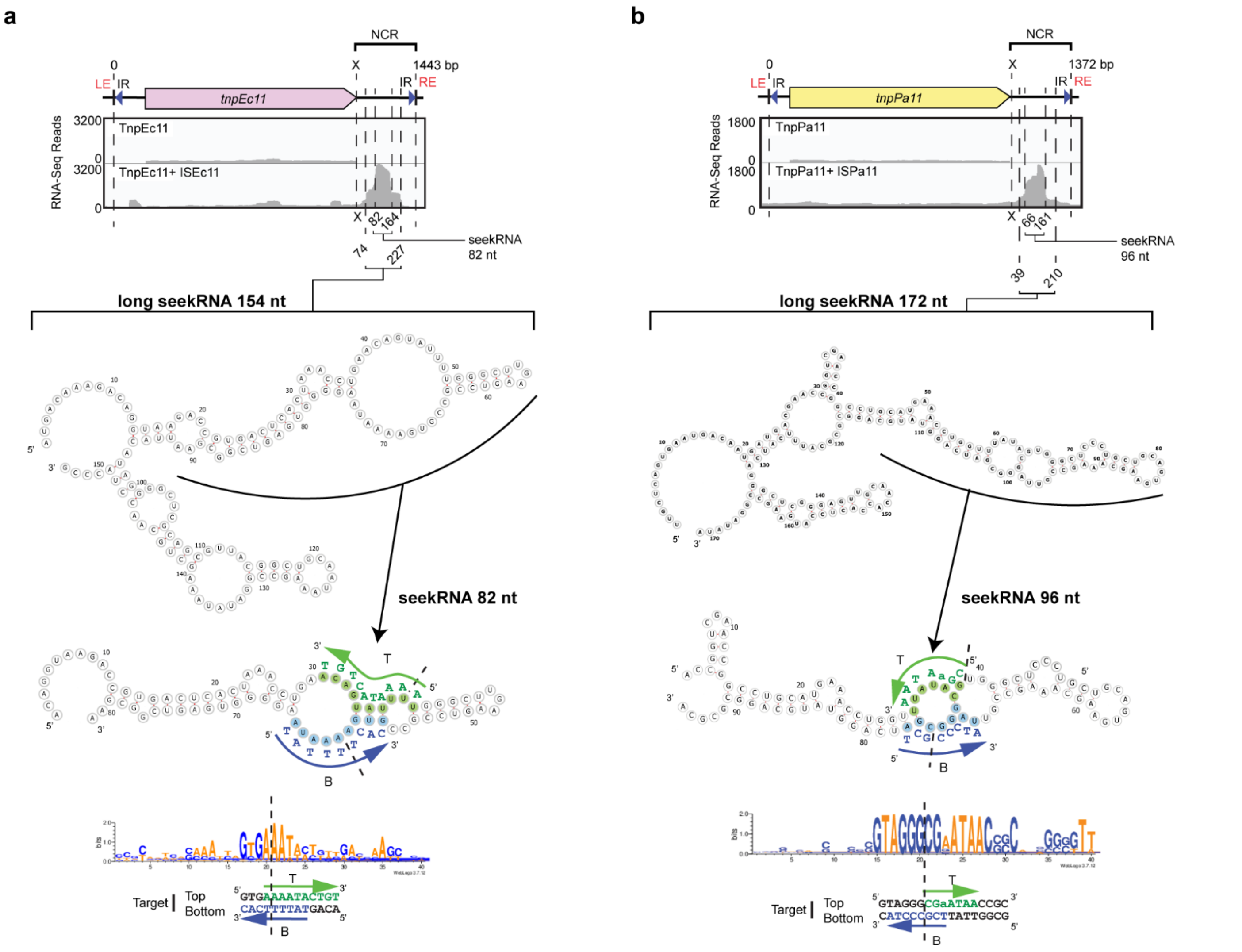

Supplementary Fig. 6: ISEc11 and ISPa11 seekRNA boundaries, folded structure and target mapping and natural reprograming of IS*1111* ISs.

RNA analysis for **a** TnpEc11-derived RNA and **b** TnpPa11-derived RNA. Small RNA-seq reads were aligned to the full length ISEc11 or ISPa11 sequence and boundaries of the RNA products shown below are measured form the *tnp* stop codon. Predicted folded structures for the long seekRNA and the seekRNA are shown below with nt matching the top and bottom strands of the corresponding target indicated in green and blue, respectively on the seekRNA and on the target sequence below. The Weblogos are also below, showing the consensus target sequences. The insertion point is marked with a dashed line on both strands of the target and the Weblogo. Inverted Repeats (IR) is represented as blue arrow and left end (LE) and right end (RE) are in red in the IS. The green arrow line indicates the top (T) DNA strand and the blue arrow line indicates the bottom (B) DNA strand of the target site. The arrow indicates the DNA polarity from 5’to 3’ end.

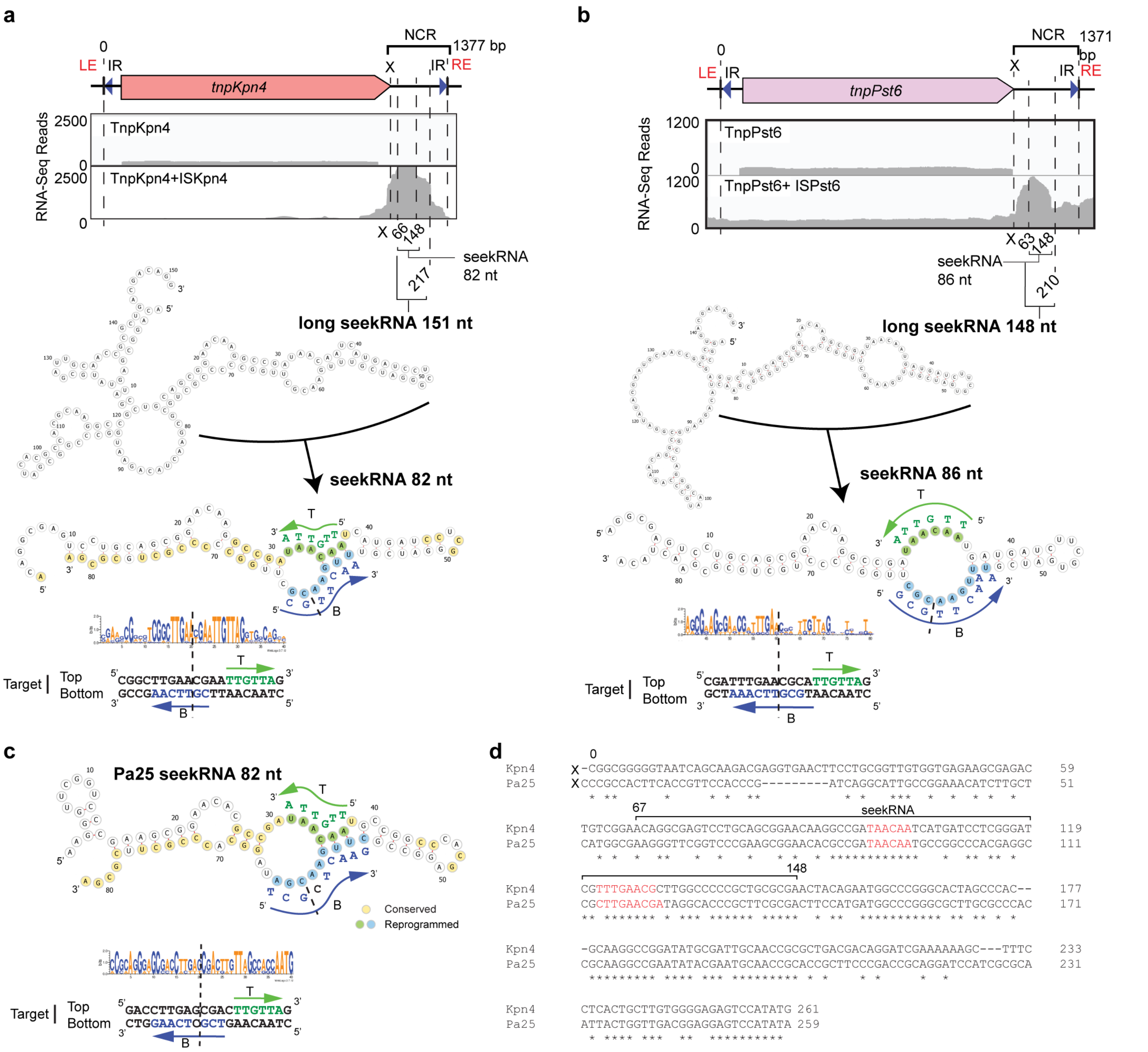

Supplementary Fig. 7: IS*1111* small RNA-seq and seek RNA folded structure similarities and differences compared with their target site

Small RNA-seq reads of **a** the TnpKpn4 expressed with the ISKpn4 present and **b** TnpPst6 expressed with ISPst6 present were aligned to the full length ISKpn4 or ISPst6 sequence and boundaries of the RNA products shown below are measured form the *tnp* stop codon. Predicted folded structures for the long seekRNA and the seekRNA are shown below with nt matching the top and bottom strands of the corresponding target indicated in green and blue, respectively on the seekRNA and on the target sequence below. The Weblogos are also below, showing the consensus target sequences. The insertion point is marked with a dashed line on both strands of the target and the Weblogo. **c** Predicted folded structure of the predicted ISPa25 seekRNA showing the same target matching residues as for the Kpn4 seekRNA. **d**, ISPa25 seekRNA was identified by alignment with the ISKpn4 NCR. In the alignment, seekRNAs are bold and bases pairing with the target site sequence are red. Transposase from ISPa25 and ISKpn4 are 46% identical. Inverted Repeats (IR) is represented as blue arrow and left end (LE) and right end (RE) are in red in the IS. The green arrow line indicates the top (T) DNA strand and the blue arrow line indicates the bottom (B) DNA strand of the target site. The arrow indicates the DNA polarity from 5’to 3’ end.

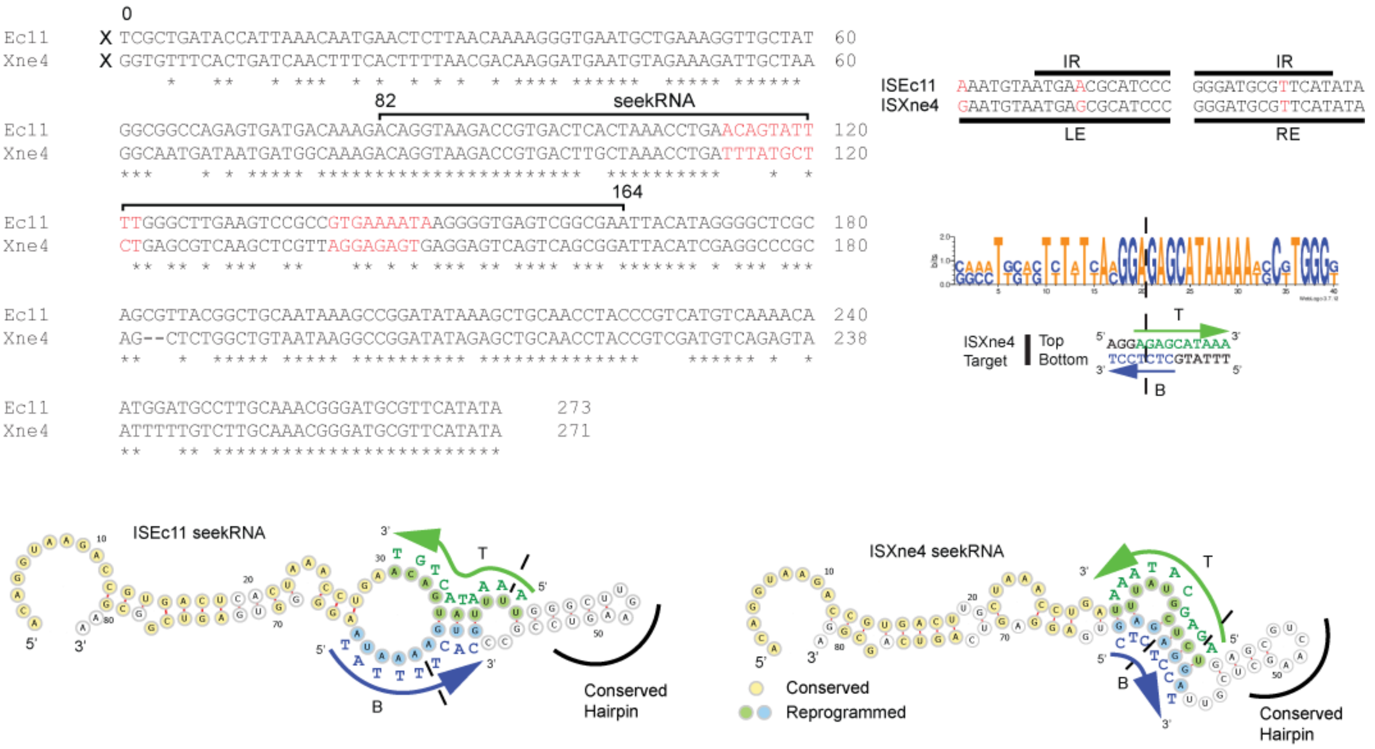

Supplementary Fig. 8: Sequence alignment the NCR region of ISEc11 and ISXne4 shows a natural redirection.

At the top, an alignment of the NCR of ISEc11 and ISXne4 starting from the stop codon of the *tnp* (x) is shown with the Weblogo for ISXne4 adjacent. Experimentally determined seekRNA for ISEc11, and predicted seekRNA for ISXne4 are marked above the alignment sequence and target matching nucleotides are red. Identical nt in the seekRNA folded structures shown below are yellow and nt matching the top (T) and bottom (B) strands of the target are green and blue respectively. The insertion point is marked with a dashed line. Transposases from ISEc11 and ISXne4 are 71.6% sequence identical. The green arrow line indicates the top (T) DNA strand and the blue arrow line indicates the bottom (B) DNA strand of the target site. The arrow indicates the DNA polarity from 5'to 3' end.

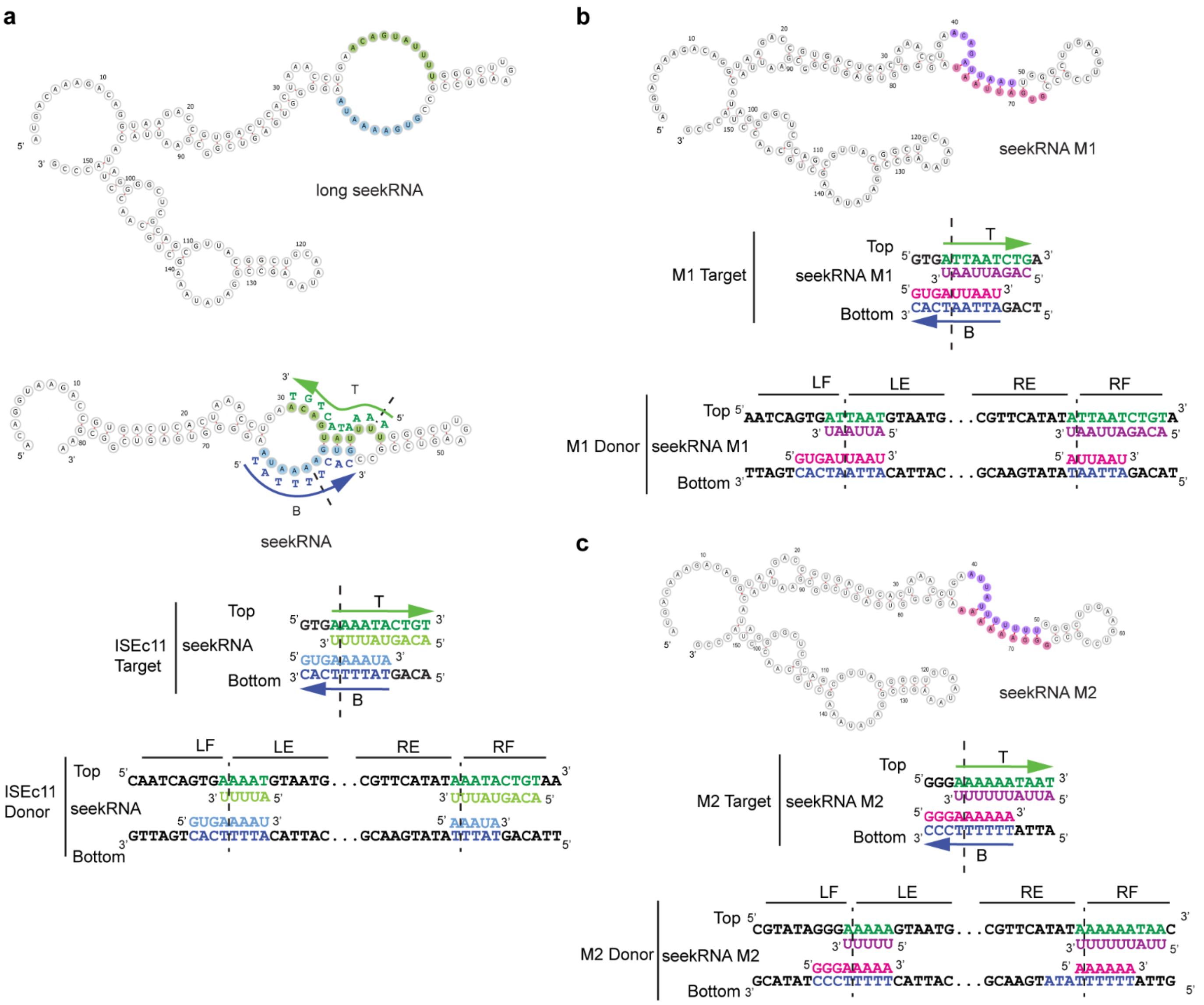

Supplementary Fig. 9: ISEc11 seekRNA modifications for reprogramming to recognize a new target

a Folded structure model of the long seekRNA and seekRNA of ISEc11 with the bases involved in base pairing with the target site sequence shown in green (top -T) strand and blue (bottom -B) strand. The DNA sequence of the target site is shown for both DNA strands with the arrow pointing towards the 3' of the corresponding strand and dashed lines indicates the insertion point of the IS. The sequence of the donor LF-LE and RE-RF of ISEc11 can be recognized by the seekRNA with base pairing nucleotides shown in green for top strand and blue for the bottom strand. **b** and **c** Folded structure model of the reprogrammed long seekRNA of ISEc11 with the bases involved in base pairing with the modified target site M1 (b) and M2 (c) showing in purple for the top (T) strand and pink for the bottom (B) strand. The corresponding sequence of the new target site M1 and M2 are shown for both DNA strands with the arrow pointing towards the 3' of the corresponding DNA strand and dashed lines indicating the insertion point of the IS. The sequence of the donor LF-LE and RE-RF of ISEc11 can be recognized by the seekRNA with base pairing nucleotides shown in green for top strand and blue for the bottom strand.

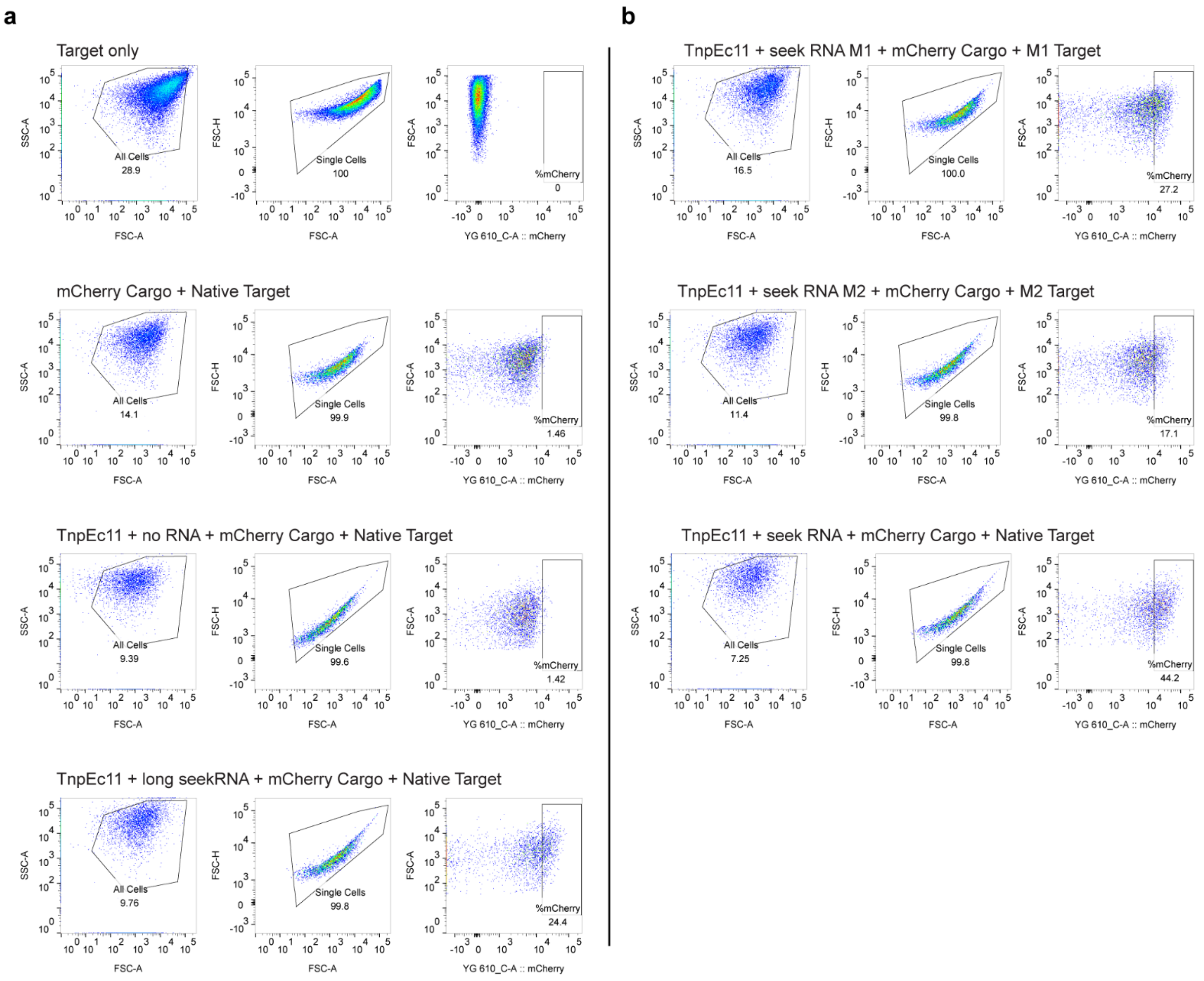

Supplementary Fig. 10: FACS gating strategy for evaluating transposition efficiency of ISEc11 seekRNA and variants using mCherry reporter.

a Cells containing different combinations of a pTarget plasmid carrying the target preceded by a T7 promoter and a pDonor carrying *tnpEc11*, ISEc11 long seekRNA and mCherry as a cargo (listed above the plots) were first gated for all cells on forward scatter area (FSC-A) and side scatter area (SSC-A) then on FSC-A and forward scatter height (FSC-H) for single cells. mCherry+ cells, indicative of transposition, were identified against background fluorescence from control samples determined. Transposition efficiency is reported as the percentage of mCherry+ cells within the single-cell population using the FSC-A and mCherry fluorescence channel at 610nm (YG 610_C-A::mCherry). **b** The approach was replicated for cells with a reprogrammed pDonor and corresponding new pTarget plasmids, or native seekRNA with native pTarget plasmids, applying identical gating criteria to quantify transposition events. Source data are provided as a Source Data file.

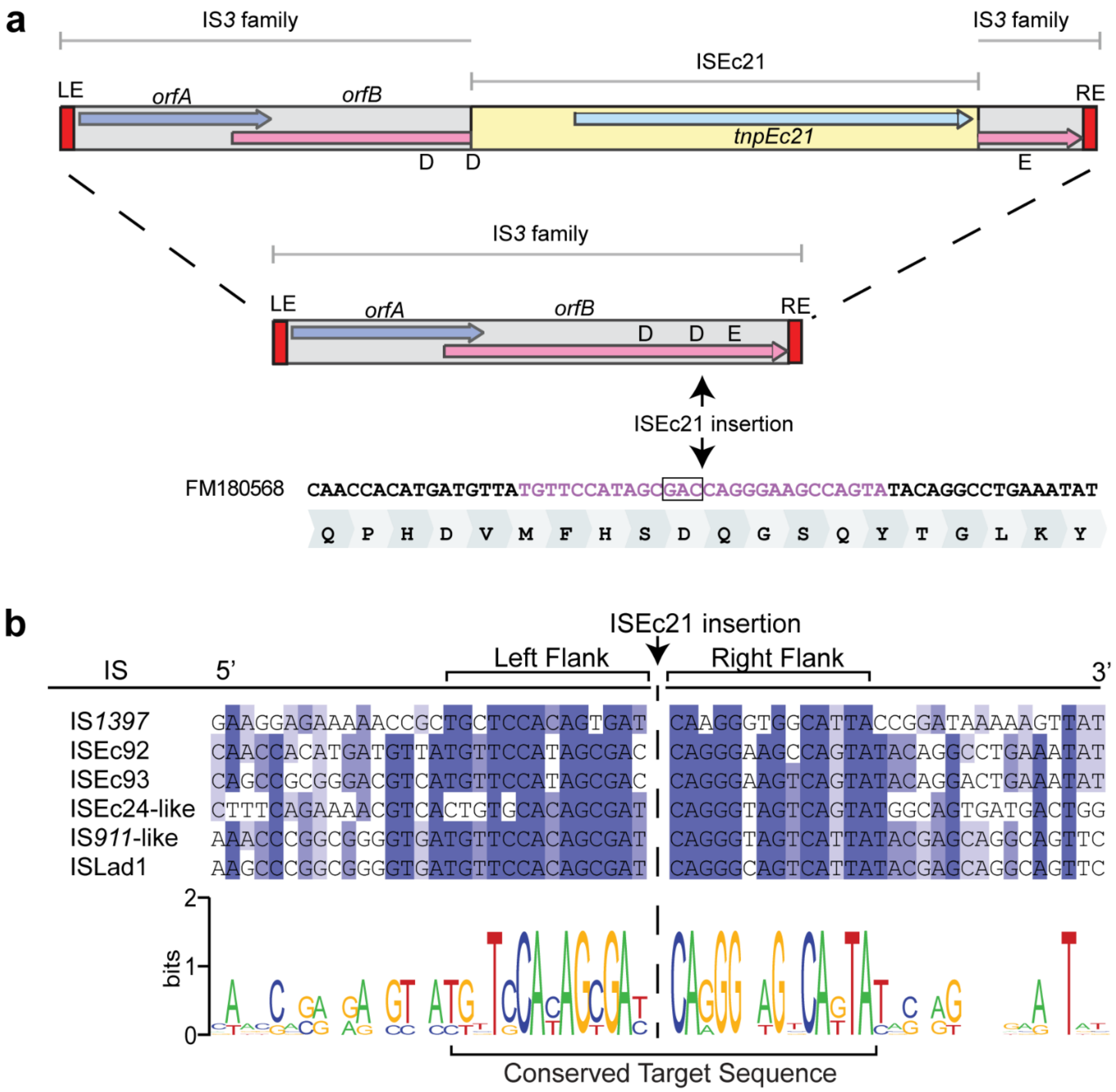

Supplementary Fig. 11: ISEc21 targets the coding sequence of DDE motif of IS*3* members.

a Schematic showing ISEc21 inserted in its target within an IS*3* family IS. Inverted repeats are red, open reading frames are arrows and the positions of codons for the DDE are shown. The codon for D is boxed in the sequence below and the vertical arrow indicates the position of the IS. **b** Alignment of the flanking sequences for ISEc21 locations in six different IS*3* family members. Arrow indicates ISEc21 insertion point. Intensity of the purple colour indicates the degree of conservation. Dark purple corresponds to 100% conserved or containing one mismatch base and no colour referring to 4 or more mismatches. The Weblogo derived from this alignment is shown below.

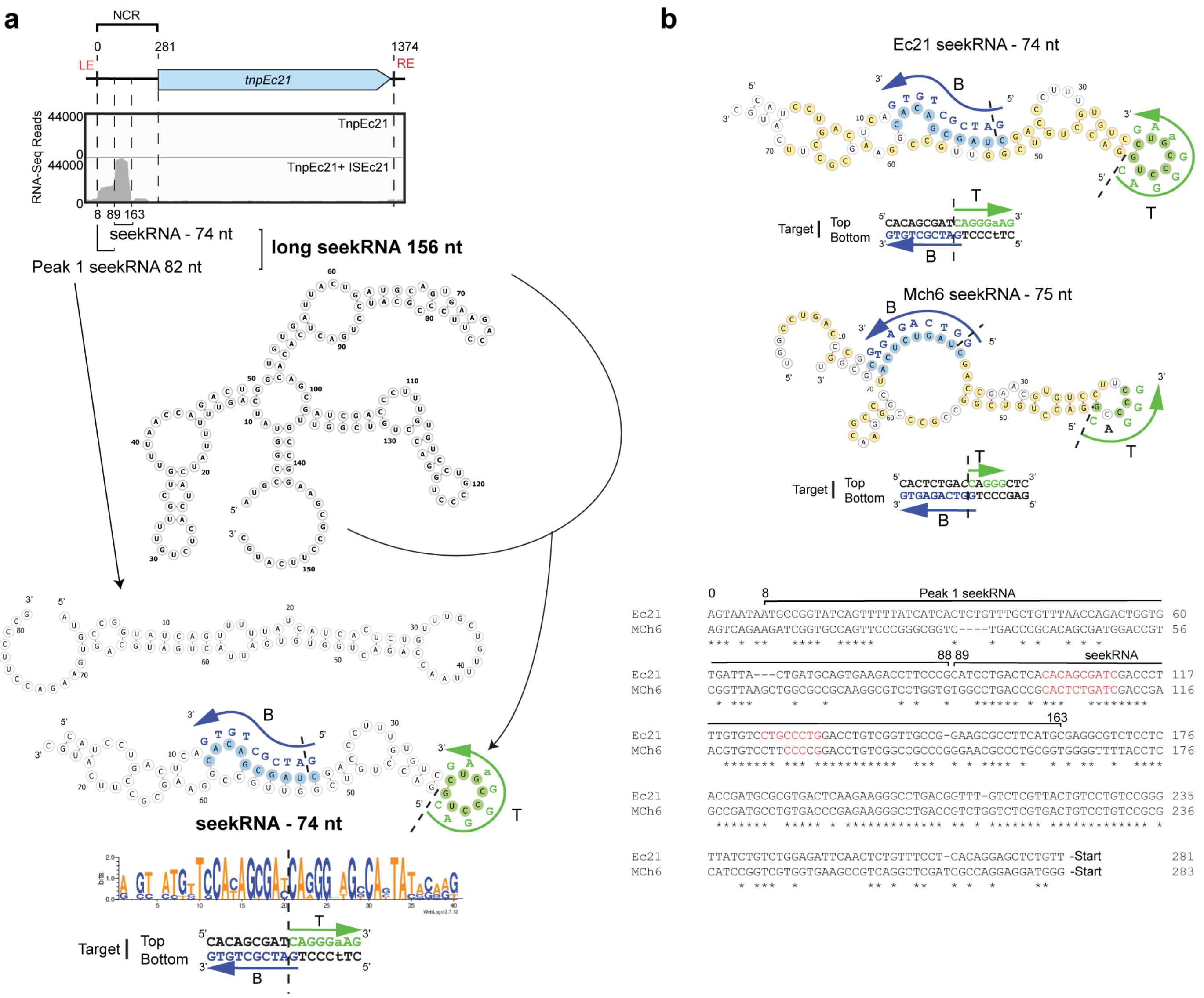

Supplementary Fig. 12: ISEc21 seekRNA folded structure and target site mapping and a naturally occurred reprogramming ISMch6

a Top panel - alignment of small RNA-seq reads for RNA associated with TnpEc21 (expressed with ISEc21) with the full length ISEc21 sequence. Below – predicted folded structures of the long seekRNA, peak 1 RNA and the seekRNA. Bottom - Consensus target sequence mapping showing both bottom and top strand matches on the seekRNA and the target sequence. **b** A predicted ISMch6 seekRNA shown folded at top was derived via a comparison of the ISEc21 and ISMch6 NCR sequences shown at bottom. The seekRNA regions are marked above the sequence with target matching regions in red. In the similar folded structures for ISEc21 and ISMch6 seekRNAs, identical bases are highlighted in yellow. The target site matches are indicated in green and blue on folded structure and target sequence. Transposases from ISEc21 and ISMch6 are 84% identical. Left end (LE) and right end (RE) are in red in the IS. The green arrow line indicates the top (T) DNA strand and the blue arrow line indicates the bottom (B) DNA strand of the target site. The arrow indicates the DNA polarity from 5’to 3’ end.

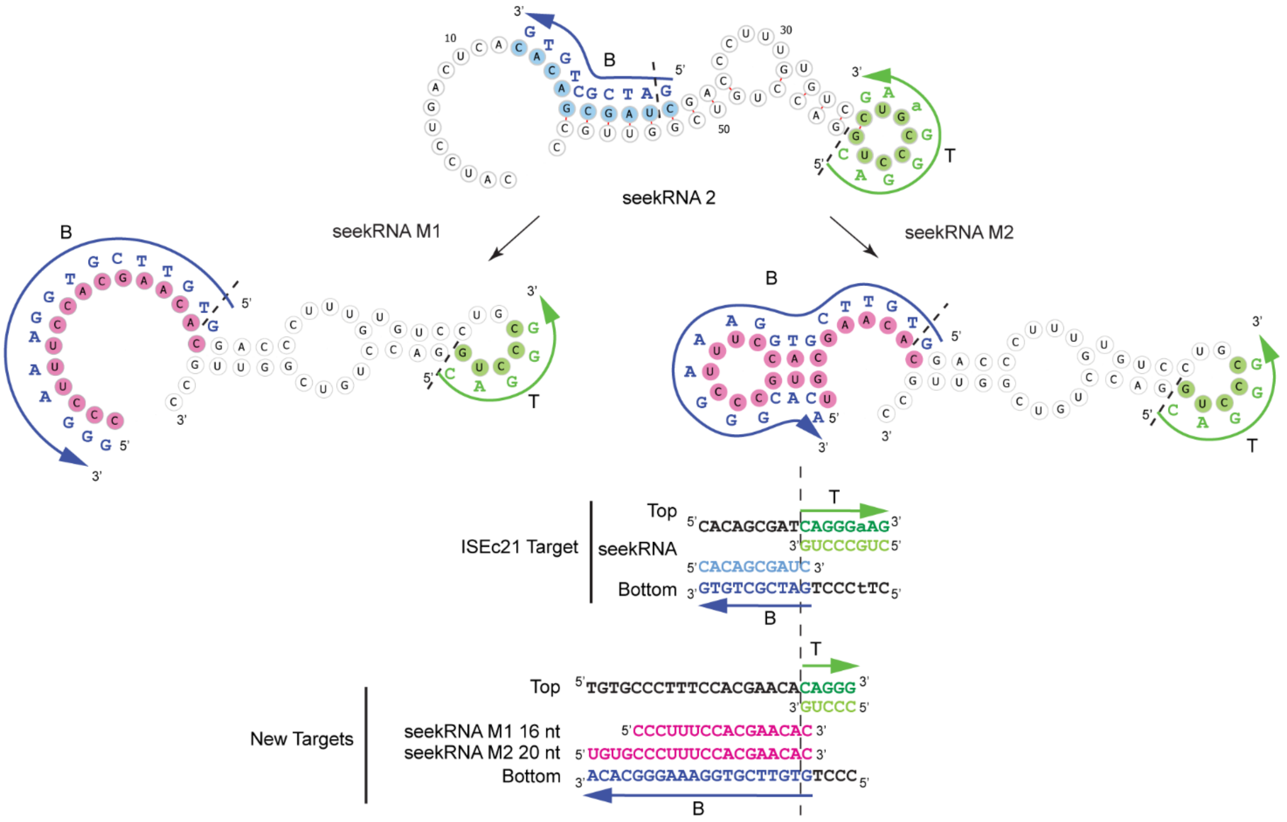

Supplementary Fig. 13: ISEc21 seekRNA modifications for reprogramming into new target site

The shorter seekRNA 2 with 58 nt (89-147) of ISEc21 folded structure is shown and the native target site mapping is shown in blue on the seekRNA. Reprograming of the shorter seekRNA 2 into M1 with addition of 15 nt to the 5'end of the short seekRNA 2, resulted in a total of 16 nt base pairing as indicated. M2 contains 4 extra nucleotides added to the 5'end of the shorter seekRNA 2 bringing the total base pairing with the target to 21 ntThe green arrow line indicates the top (T) DNA strand and the blue arrow line indicates the bottom (B) DNA strand of the target site. The arrow indicates the DNA polarity from 5'to 3' end.

Supplementary Table 1. Sequences of the NCRs present in the ISs used in this study.

IS family	IS Name	NCR and IS ends *	seekRNA size (nt)
IS*1111*	ISEc11	ACGCTGATACCATTAAACAATGAACTCTTAACAAAAGGGTGAATGCTGAAAGGTTGCTATGGCGGCCAGAGTGATGACAAAGACAGGTAAGACCGTGACTCACTAAACCTGAACAGTATTTTGGGCTTGAAGTCCGCCGTGAAAATAAGGGGTGAGTCGGCGAATTACATAGGGGCTCGCAGCGTTACGGCTGCAATAAAGCCGGATATAAAGCTGCAACCTACCCGTCATGTCAAAACAATGGATGCCTTGCAAACGGGATGCGTTCATATA	82
IS*1111*	ISKpn4	GGGCGGGGGTAATCAGCAAGACGAGGTGAACTTCCTGCGGTTGTGGTGAGAAGCGAGACTGTCGGAACAGGCGAGTCCTGCAGCGGAACAAGGCCGATAACAATCATGATCCTCGGGATCGTTTGAACGCTTGGCCCCCGCTGCGCGAACTACAGAATGGCCCGGGCACTAGCCCACGCAAGGCCGGATATGCGATTGCAACCGCGCTGACGACAGGATCGAAAAAAGCTTTCCTCACTGCTTGTGGGGAGAGTCCATATG	82
IS*1111*	ISPst6	CGGCGAGGTAAATAATGAGGTGAGTGTCTAGTGGTTGTGGTGAGAAGCGAGACTGTCGGAACAGGCGAGTCCTGCAGCGGAACAAGGCCGATAACAATGATGATCTTCGTGATCGTTTGAACGCTTGGCCCCTGCTGCGCGAACTACAGAATGGCCAGGGCAATGCCCAGAACAGGCCGGATATACGAATGCAACCGCGCTGACGACAGGATCGAAAAAAGCTATTCCTCACTGCTTGTGGGGAGAGTCCATATA	86
IS*1111*	ISPa11	TCGTTCCTGCCACACCGTAGTTGAAACATCCACCACGATTGCTCAGTGAATGACAATGATGACGAACCGGTCGAACCGGCCTGCATGAAACCTGGTTTATACGTGGGCTCCCTGCTGCAGTGAAGCAAAGCCGTTAGGGCGATCAGGTATGCAGGCGCGCATTTCATCAGGGCTCGGGAGTTGCAACACCACTCCATGAAGCCGGATATACGGATGCAGTCGTACACAGGTTTGAAATCAAGACAAACACTGGCAAACCGGGAGGAGTCCATATA	96
IS*110*	ISEc21	AGTAATAATGCCGGTATCAGTTTTTATCATCACTCTGTTTGCTGTTTAACCAGACTGGTGTGATTACTGATGCAGTGAAGACCTTCCCGCATCCTGACTCACACAGCGATCGACCCTTTGTGTCCTGCCCTGGACCTGTCGGTTGCCGGAAGCGCCTTCATGCGAGGCGTCTCCTCACCGATGCGCGTGACTCAAGAAGGGCCTGACGGTTTGTCTCGTTACTGTCCTGTCCGGGTTATCTGTCTGGAGATTCAACTCTGTTTCCTCACAGGAGCTCTGTT	74

* The seekRNA is in blue, the long seekRNA is marked with gray background, the IR of IS*1111* family IS and IS ends are green.

Supplementary Table 2. Outward facing primers used to detect mini-circle formation

**Primer name**	**Sequence (5' to 3')**	**Figures**
SFA08-F Ec11	TAACCGCCCATGACCGTAAG	Fig 2a
SFA07-R Ec11	CTCACGGTTGCCGAAGAACG	Fig 2a
SFA22-F Ec21	CGTTATCGACTTAACCGGGG	Fig 6a, e
SFA21-R Ec21	ACAGATAACCCGGACAGGAC	Fig 6a, e

Supplementary Table 3. Primers used to detect transposition.

**Primer name**	**Description**	**Sequence (5' to 3')**	**Figures**
SFA188-F pRSF	Primer in pRSF	AACACAATCTTCCTGCTCAG	Fig 2c-d, 4b-c, 6c, 7b-d
SFA184-R pRSF	Primer in pRSF	AGTGGTGCTGTTACTTAC	Fig 2c, 6c
SFA08-F Ec11	Primer in *tnpEc11*	TAACCGCCCATGACCGTAAG	Fig 2c
SFA190-R Ec11	Primer in *tnpEc11*	AAATCCTGTCTACGCAACTG	Fig 2c-d
SFA97-F Target	Primer in pRSF	TCTCCGGGCATATCTTCGTC	Fig 5a
SFA55-R Ec11	Primer in *tnpEc11*	TTAATATGGTCTGATACTGACGT	Fig 5a
SFA30-F Cat	Primer in *catA1*	TTCCAACTTTCACCATAATGAAATAAGATCACTACCG	Fig 5b
SFA148-R pRSF	Primer in pRSF	TGCAGCGCTTGACTTGAC	Fig 5b
SFA21-R Ec21	Primer 38 bp upstream of *tnpEc21*	ACAGATAACCCGGACAGGAC	Fig 6c
SFA22-F Ec21	Primer in *tnpEc21*	CGTTATCGACTTAACCGGGG	Fig 6c
SFA162-F Ec21	Primer in *tnpEc21*	GAACCGTCACACCCAAAACG	Fig 6e
SFA96-R pRSF	Primer in pRSF	CGTTTCCCGTTGAATATGGC	Fig 6e
SFA270-R mCherry	Primer in *mCherry*	TAAGCACCCGGTAACTGTAC	Fig 4b-c, 7b-d
SFA213-R mCherry	Primer in *mCherry*	ACTCTTTAATGATCGCCATG	Fig 4b-c, 7b-d
SFA214-F mCherry	Primer in *mCherry*	CCGGTGGTATGGATGAACTG	Fig 4b-c, 7b-d
SFA189-R Target pRSF	Primer in pRSF	AATATAGGATCGGACAGCCC	Fig 4b-c, 7b-d

Genes are in italics
