# Supplementary material for: A programmable seekRNA guides target selection by IS1111 and IS110 type insertion sequences
Source: Nat Commun. 2024 Jun 19;15:5235. doi: 10.1038/s41467-024-49474-9 (PMC11187229; doi:10.1038/s41467-024-49474-9)
Supplement: Supplementary file 5 — Reporting Summary [file 41467_2024_49474_MOESM5_ESM.pdf]

nature portfolio

Corresponding author(s): Sanddro F Ataide

Last updated by author(s): Jun 5, 2024

Reporting Summary

Nature Portfolio wishes to improve the reproducibility of the work that we publish. This form provides structure for consistency and transparency in reporting. For further information on Nature Portfolio policies, see our Editorial Policies and the Editorial Policy Checklist.

Statistics

For all statistical analyses, confirm that the following items are present in the figure legend, table legend, main text, or Methods section.

n/a	Confirmed	
☐	☒	The exact sample size (*n*) for each experimental group/condition, given as a discrete number and unit of measurement
☐	☒	A statement on whether measurements were taken from distinct samples or whether the same sample was measured repeatedly
☒	☐	The statistical test(s) used AND whether they are one- or two-sided<br>*Only common tests should be described solely by name; describe more complex techniques in the Methods section.*
☒	☐	A description of all covariates tested
☒	☐	A description of any assumptions or corrections, such as tests of normality and adjustment for multiple comparisons
☐	☒	A full description of the statistical parameters including central tendency (e.g. means) or other basic estimates (e.g. regression coefficient) AND variation (e.g. standard deviation) or associated estimates of uncertainty (e.g. confidence intervals)
☒	☐	For null hypothesis testing, the test statistic (e.g. *F*, *t*, *r*) with confidence intervals, effect sizes, degrees of freedom and *P* value noted<br>*Give P values as exact values whenever suitable.*
☒	☐	For Bayesian analysis, information on the choice of priors and Markov chain Monte Carlo settings
☒	☐	For hierarchical and complex designs, identification of the appropriate level for tests and full reporting of outcomes
☒	☐	Estimates of effect sizes (e.g. Cohen's *d*, Pearson's *r*), indicating how they were calculated

Our web collection on statistics for biologists contains articles on many of the points above.

Software and code

Policy information about availability of computer code

Data collection	Illumina iSeq100 System, TECAN infinite M1000Pro plate reader, BD LSRFortessa™ X-20 Flow Cytometer. All custom code developed for this manuscript is available at https://github.com/ataidelab/targets. Additionally, a DOI has been created, doi.org/10.5281/zenodo.11435551
Data analysis	Microsoft Excel v2405 (Build 17628.20110), Flowjo v10.10, GraphPad Prism v10.1.2, MEGA v11, Clustal Omega, Weblogo, Alphafold2, Pymol v1.8, Blast+ v2.13.0, BWA 0.7.18, IGV v2.17.1, Tablet v1.21.02.08, MXfold2, seqtk, perl, python, python packages (biopython, bio, weblogo)

For manuscripts utilizing custom algorithms or software that are central to the research but not yet described in published literature, software must be made available to editors and reviewers. We strongly encourage code deposition in a community repository (e.g. GitHub). See the Nature Portfolio guidelines for submitting code & software for further information.

Data

Policy information about availability of data

All manuscripts must include a data availability statement. This statement should provide the following information, where applicable:

- Accession codes, unique identifiers, or web links for publicly available datasets
- A description of any restrictions on data availability
- For clinical datasets or third party data, please ensure that the statement adheres to our policy

Genbank genomes and annotated IS from ISFinder were used to generate the GitHub are publicly available. Data to support the results are in the main text and the

Supplementary Information and Source Data. Small RNA-Seq data from Illumina sequencing datasets generated in this study are available on NCBI Sequence Read Archive under PRJNA1091059 (https://www.ncbi.nlm.nih.gov/sra/PRJNA1091059). Additional data will be made available upon reasonable request.

Research involving human participants, their data, or biological material

Policy information about studies with human participants or human data. See also policy information about sex, gender (identity/presentation), and sexual orientation and race, ethnicity and racism.

Reporting on sex and gender	N/A
Reporting on race, ethnicity, or other socially relevant groupings	N/A
Population characteristics	N/A
Recruitment	N/A
Ethics oversight	N/A

Note that full information on the approval of the study protocol must also be provided in the manuscript.

Field-specific reporting

Please select the one below that is the best fit for your research. If you are not sure, read the appropriate sections before making your selection.

☒ Life sciences ☐ Behavioural & social sciences ☐ Ecological, evolutionary & environmental sciences

For a reference copy of the document with all sections, see nature.com/documents/nr-reporting-summary-flat.pdf

Life sciences study design

All studies must disclose on these points even when the disclosure is negative.

Sample size	All experiments described were performed for the first time. For the manuscript, biological replicate was performed and PCR, plate reader and FACS to detect transposition was performed for each replicate with excellent reproducibility.
Data exclusions	there is no data exclusion
Replication	all experiments were performed with separate experimental biological triplicate for transposition and minicircle detection. For plate reader and FACS, 3 biological replicate of the cells were transformed with corresponding plasmids and measured separately.
Randomization	no randomization was used in the study
Blinding	all the experiments were performed by RS and SFA analyzed the data separatedly

Reporting for specific materials, systems and methods

We require information from authors about some types of materials, experimental systems and methods used in many studies. Here, indicate whether each material, system or method listed is relevant to your study. If you are not sure if a list item applies to your research, read the appropriate section before selecting a response.

Materials & experimental systems

n/a	Involved in the study	
☒	☐	Antibodies
☒	☐	Eukaryotic cell lines
☒	☐	Palaeontology and archaeology
☒	☐	Animals and other organisms
☒	☐	Clinical data
☒	☐	Dual use research of concern
☒	☐	Plants

Methods

n/a	Involved in the study	
☒	☐	ChIP-seq
☐	☒	Flow cytometry
☒	☐	MRI-based neuroimaging

Plants

Seed stocks: N/A

Novel plant genotypes: N/A

Authentication: N/A

Flow Cytometry

Plots

Confirm that:

☒ The axis labels state the marker and fluorochrome used (e.g. CD4-FITC).

☒ The axis scales are clearly visible. Include numbers along axes only for bottom left plot of group (a 'group' is an analysis of identical markers).

☒ All plots are contour plots with outliers or pseudocolor plots.

☒ A numerical value for number of cells or percentage (with statistics) is provided.

Methodology

Sample preparation: 100 ng of each plasmid (pDonor and pTarget) (Supplementary Table 3) was co-transformed into E. coli BL21 (DE3) cells via electroporation. Cells were plated on fresh agar plates containing Spectinomycin, Kanamycin and 0.1 mM IPTG to induce expression of Transposase and seekRNA, as well as expression of mCherry after transposition. The plates were incubated for 16 hours at 37 °C, followed by 4 hours at room temperature. Entire agar plates were scraped containing hundreds of colonies and resuspended and mixed evenly in 1 mL of LB media. Cells were diluted 1 in 2 in PBS (Phosphate Buffered Saline, pH 7.4) and run on the Flow Cytometer.

Instrument: BD LSRFortessa™ X-20 Flow Cytometer

Software: FlowJo™ v10.10 Software

Cell population abundance: Around 35000-70000 cells were run for each sample until at least 25000 events were recorded, which were gated for single live cells.

Gating strategy: Cells co-transformed with pDonor and pTarget plasmids carrying ISEc11 seekRNA full and mCherry as a cargo (listed above the plots) were first gated for all cells on FSC-A and SSC-A then on FSC-A and FSC-H for single cells. mCherry+ cells, indicative of transposition, were identified against background fluorescence from control samples. Transposition efficiency is reported as the percentage of mCherry+ cells within the single-cell population. Each set of samples was created by three independent transformations as biological repeats, and the transposition frequency was plotted as bar graphs. (Supplementary Fig 10) and Source data avaialbe for the cell counting for each independent biological replicate

☒ Tick this box to confirm that a figure exemplifying the gating strategy is provided in the Supplementary Information.
